# Early death in pediatric cancer: remaining questions and next steps

**DOI:** 10.18632/oncotarget.22257

**Published:** 2017-11-01

**Authors:** Elissa Furutani, Carlos Rodriguez-Galindo, Adam L. Green

**Affiliations:** Adam L. Green: Children’s Hospital Colorado/University of Colorado School of Medicine, Aurora, CO, USA

**Keywords:** child, neoplasms, healthcare disparities, socioeconomic factors, SEER program

Pediatric cancer outcomes have improved significantly over the past several decades; however, a group of patients still die very early in their disease course. Patients may have severe complications from their disease by the time they present to care, or have early complications from treatment. Some of these patients are too ill to be able to participate in clinical trials, thus this group is not well characterized in the literature. Understanding more about this population may lead to improved outcomes for all childhood cancer patients.

We performed a retrospective analysis on 36,337 cancer patients ages 0-19 years old using 13 SEER registries from 1992-2011 [1]. Early death, which we defined as a death within a month of diagnosis, did not affect all diagnoses, demographics, or socioeconomic groups equally. These children were more likely to be younger (< 1 year of age) and of black race or Hispanic ethnicity. Those with early death and hematologic malignancies were more likely to live in low-income counties. Certain tumor types were over-represented, including AML, infant ALL, hepatoblastoma, and malignant brain tumors. Although the percentage of early deaths declined over time (APC -2.5; 95% CI, -3.9 to -1.1), the incidence of early deaths is still high (1.5% overall incidence, representing 7.5% of total deaths in the cohort), especially when compared to published clinical trial data. Compared to SEER-derived, cancer subtype-matched data from the same time period, cooperative group clinical trials reported early death rates ranging from 50% to more than 90% lower [1].

This cohort is the largest to date describing early death in childhood cancer. However, there are multiple limitations to this study. One is the retrospective nature, and due to the coding of data within SEER, we cannot parse out when death occurred during that first month. The reason for death is not fully captured in this dataset. In addition, data from these registries may not be fully representative of the United States, or other countries, both with regards to socioeconomic status and access to care.

Two previous studies looking at this population have identified overlapping risk factors for early death, including young age and certain cancer types; these prior studies did not identify demographic or socioeconomic factors as a risk factors [2, 3].However, there has been increasing interest in the effect of socioeconomic disparities on cancer outcomes, with lower socioeconomic status correlated with worse outcomes and advanced stage at diagnosis [4, 5]. Although there are many efforts underway to improve cancer care in low income countries, we and others note that these disparities can also exist within high-income countries [6].

The reasons for disparities within this group of is likely multifactorial. What remains unclear are the relative contributions of ethnic and racial differences in cancer biology, access to care, and quality of care. Previous studies have started to investigate the biology of disparities in pediatric acute lymphoblastic leukemia [7]. Others demonstrated an association between uninsured status and poor outcomes [6, 8]. To our knowledge, there has not been a study which has compared the early death rates in countries with universal health care to those with systemic healthcare disparities. Parsing out these issues is particularly important as access to care will be greatly affected by policies currently being debated within our country.

Other questions also remain about this population at risk. The early death rate in this study is much higher than previously reported in clinical trials, likely due to ineligibility of patients with late stage disease or critical illness at the time of presentation for these trials. One way of prospectively gathering data on this cohort includes cooperative group efforts such as the Children’s Oncology Group (COG) Project Every Child, which gathers not only demographic, treatment, and outcome data, but also links these data to tissue samples which may serve to identify biological factors that may predispose to more aggressive disease. We are developing a companion COG study to interview parents of early death patients to better understand the factors involved with this phenomenon at the patient and family level.

Pediatric cancer treatment has made many strides over the past 50 years, and it is reassuring that there has been a decline in early death rates over the period of our study. This could be due to a combination of improved diagnostic capability, improvement in supportive care, and more effective therapies, and there are tremendous efforts underway to continue to improve upon these. However, it is clear that not all groups of patients have benefited equally from these advances, and we hope that understanding the reason for these continued disparities may lead to more equity in application of these improvements to all children.
